# A Novel Temperature Drift Error Precise Estimation Model for MEMS Accelerometers Using Microstructure Thermal Analysis

**DOI:** 10.3390/mi13060835

**Published:** 2022-05-26

**Authors:** Bing Qi, Shuaishuai Shi, Lin Zhao, Jianhua Cheng

**Affiliations:** 1 College of Intelligent Systems Science and Engineering, Harbin Engineering University, Harbin 150001, China; shishuaishuai96@163.com (S.S.); zhaolin@hrbeu.edu.cn (L.Z.); chengjianhua@hrbeu.edu.cn (J.C.); 2 JONHON, Luoyang 471003, China

**Keywords:** MEMS accelerometer, temperature dependence, microstructure thermal analysis, TDE precise test based on heat conduction analysis, PSO-GA-BPNN

## Abstract

Owing to the fact that the conventional Temperature Drift Error (TDE) precise estimation model for a MEMS accelerometer has incomplete Temperature-Correlated Quantities (TCQ) and inaccurate parameter identification to reduce its accuracy and real time, a novel TDE precise estimation model using microstructure thermal analysis is studied. First, TDE is traced precisely by analyzing the MEMS accelerometer’s structural thermal deformation to obtain complete TCQ, ambient temperature *T* and its square *T*^2^, ambient temperature variation ∆*T* and its square ∆*T*^2^, which builds a novel TDE precise estimation model. Second, a Back Propagation Neural Network (BPNN) based on Particle Swarm Optimization plus Genetic Algorithm (PSO-GA-BPNN) is introduced in its accurate parameter identification to avoid the local optimums of the conventional model based on BPNN and enhance its accuracy and real time. Then, the TDE test method is formed by analyzing heat conduction process between MEMS accelerometers and a thermal chamber, and a temperature experiment is designed. The novel model is implemented with TCQ and PSO-GA-BPNN, and its performance is evaluated by Mean Square Error (MSE). At last, the conventional and novel models are compared. Compared with the conventional model, the novel one’s accuracy is improved by 16.01% and its iterations are reduced by 99.86% at maximum. This illustrates that the novel model estimates the TDE of a MEMS accelerometer more precisely to decouple temperature dependence of Si-based material effectively, which enhances its environmental adaptability and expands its application in diverse complex conditions.

## 1. Introduction

As natural resource exploration turns from the earth’s surface to deep space, instead of human beings, unmanned intelligent devices are widely used in the harsh deep space environment. Rovers, as its typical representative, carry all diverse kinds of devices for missions, and stability is the key factor. Before launching them to target planets, the terrain has to be investigated as comprehensively as possible to ensure that they will not be in danger and can complete their missions successfully. However, owing to the fact that the extraterrestrial environment is complex and changeable, it is very hard to obtain topographic information as accurately as true, which especially makes terrain pre-investigation unpredictable. For example, rovers can get stuck in dust, causing them to roll over and crash. Therefore, it is essential for unmanned intelligent devices to dispose of the risks autonomously and intelligently [1].

Three-dimensional status measurement shows the stability of unmanned intelligent devices, and they take some measures to respond to the risks. For example, rovers use emergency braking to avoid rolling over and fast reverse to avoid collision. Therefore, 3D status measurement plays a role in stability [2,3,4]. As it is known, rocket load is limited, so MEMS accelerometers of smaller size and lighter weight are a better choice for unmanned intelligent devices. They are made up from Si-based material of temperature dependence, and ambient temperature changes their physical properties to excite TDE. Ambient temperature in space is −180 °C~130 °C, which inevitably worsens their output consistency and accuracy. Therefore, TDE seriously restricts the universal application of MEMS accelerometers [5].

Due to current limitations in material processing, Si-based material is a better choice to manufacture MEMS accelerometers, but it is unlikely to eliminate its temperature dependence and compensate TDE by optimizing the production process [6]. Therefore, common methods to compensate TDE include hardware calibration and algorithm compensation [7,8,9]. The first one utilizes a temperature control system to maintain ambient temperature stably in a narrow range, which stabilizes its internal structure relatively [10,11]. Although it works with a perfect seal structure and heat conduction, high-power consumption and complex thermal noise are difficult problems to solve [12,13]. By comparison, algorithm compensation has high calculation accuracy and perfect implementation, and TDE can be estimated with a specific algorithm without additional devices, precisely and in real time [14]. Therefore, it is the mainstream of TDE estimation for MEMS accelerometers, and TDE traceability and its implementation are its important parts.

TDE traceability is the most concentrated method to improve the accuracy of MEMS accelerometers by theoretical derivation [15]. Reference [16] concludes ambient temperature is a direct cause for TDE, and also proves ambient temperature variation of 1 °C changes quality factor’s sensitivity up to 1% by experiments. However, root causes exciting TDE have not been studied completely. Reference [17] builds a simulation model to comprehensively research the multi-factor coupling behavior of MEMS accelerometers under high-temperature impact load. It shows the key to TDE traceability is a deep exploration of its thermal characteristics. From the relationship between its structure and ambient temperature, reference [18] shows that the physical sizes of Si-based materials expand linearly as ambient temperature in 3D space, which deforms its internal microstructure and deviates its performances. A comprehensive TDE estimation model is built based on the relationship above, and its bias stability is enhanced by 10% from before. Reference [19] presents multi-physical analysis to research TDE. Using the finite element method in simulation, the factor to TDE is a mismatch of Coefficient of Thermal Expansion (CTE) between different materials. Moreover, it illustrates that a deviation between simulations and experiments is about 1~2 mg/°C, which means CTE is non-ignorable to TDE traceability. Then, it completes many tests on physical characteristics of Si-based material, which shows Thermal Expansion Properties (TEP) of Si-based material are nonlinear from a global perspective. Hence, there must be remnant TDE to be uncompensated comprehensively, and it is essential to trace TDE more accurately and necessary to research TCQ of MEMS accelerometers more precisely.

After obtaining accurate TDE traceability, describing the relationship between TCQ and TDE precisely is another key factor, and the TDE estimation model is accurate and efficient. Establishing the TDE estimation model accurately focuses on the relationship between its input and output by algorithm, and the outputs of MEMS accelerometers are compensated to the desired ones with its inputs [20]. Reference [21] proposes a modified Support Vector Machine (SVM) model with a structural risk minimization principle, and PSO is introduced in optimizing SVM and increasing the MEMS accelerometer’s output accuracy. Even with small-batch data processing, it also maintains its performance. By comparing with the conventional SVM, PSO tuning SVM improves its output accuracy by 18.96%, 16.65%, and 14.53%, respectively, in three tests. Due to its complexity, SVM is unsuitable to process a lot of test results and cannot increase calculation accuracy and real time. Reference [22] builds a wavelet neural network model based on large quantities of results from MEMS gyros, and its attitude accuracy can be improved to 8arcsec after TDE is compensated. However, it is very hard to identify the structure parameters and obtain the optimal values because of the diversity and uncertainty of its internal signal transmission. Reference [23] proposes a TDE estimation model for MEMS accelerometers using a language model back propagation neural network. Its parameters are determined by optimizing the compensation model, and maximum nonlinearity decreases from 3329 ppm to 603 ppm. Owing to the fact that BPNN has the demerits of local minimums, it may easily obtain some non-optimal results to reduce its TDE estimation accuracy. Reference [24] proposes a TDE estimation model based on BPNN optimized by GA. GA is introduced to assist in searching the optimal values, which avoids BPNN to have local minimums. After being compensated, the maximum bias of the MEMS accelerometer is 0.017% over −10 °C~80 °C, and it is 173-times more accurate than the conventional TDE estimation models based on polynomial fitting. However, GA has probabilistic disorder to reduce its calculation real time. Hence, it is necessary to enhance TDE calculation real time, as well as maintaining TDE estimation accuracy.

In the paper, TDE traceability for a MEMS accelerometer is researched with microstructure thermal analysis by simulating its structural deformation in diverse conditions, and new TRQ are deduced as the key factor to TDE traceability. Then, a novel TDE precise estimation model for the MEMS accelerometer is established based on *T* and *T*^2^, as well as ∆*T* and ∆*T*^2^. To increase TDE estimation accuracy and real time, GA is introduced in the conventional model to remove local optimums of BPNN. Further, PSO is utilized in GA to solve its probabilistic disorder to improve its TDE calculation rapidity. The novel model estimates TDE more precisely, which decouples temperature dependence of Si-based materials better and more effectively to enhance environmental adaptability of MEMS accelerometers. The novel model is significant to expand its application in diverse complex conditions and ensures the accuracy and stability of unmanned intelligent devices.

## 2. Methodology

### 2.1. TDE Estimation Model Establishment

#### 2.1.1. Conventional Estimation Model for TDE

**Figure 1:**
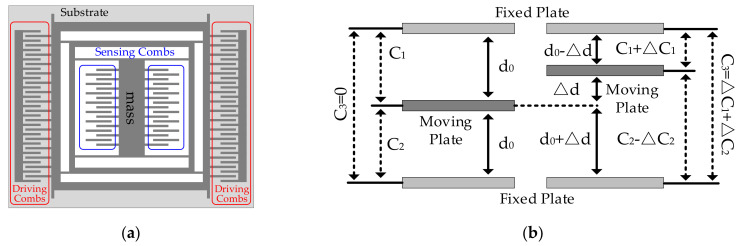
Principle of MEMS accelerometer. (**a**) hardware design; (**b**) system schematic diagram.

MEMS accelerometer is a miniaturized device made up from Si-based material, including mass, driving circuit, sensing circuit, and substrate. With a series of manufacturing procedures, they are assembled as a micromachining unit. Figure 1 shows its principle [18].

From Figure 1, sensing circuit and driving circuit have combs, and all the combs can be seen as plate capacitors. The combs of mass are a moving plate, and the combs of substrate are a fixed plate. Therefore, the carriers’ acceleration is obtained by measuring the capacitance variation between the combs [18]. When the carriers stay still, the moving plates are in balance. With plate capacitor formula, the capacitance of fixed plate and moving plate is shown:

(1) $$C_{1}=C_{2}=\frac{\varepsilon }{4\pi k}\frac{S_{0}}{d_{0}}=C_{0}$$

where, *ε* is relative dielectric constant, *k* is electrostatic force constant, *s*_0_ is overlap area of moving and fixed plates. From (1), the capacitance *C*_3_ between the fixed plates is shown:

(2) $$C_{3}=\left|C_{1}-C_{2}\right|=0$$

When the carriers accelerate or slow down, a displacement appears between the mass and the sensing circuit under the Coriolis force, then *C*_3_ is shown as follows:

(3) $$C_{3}=\left|C_{1}-C_{2}\right|=\left|\frac{\varepsilon }{4\pi k}\frac{S_{0}}{\left(d_{0}-\Delta d\right)}-\frac{\varepsilon }{4\pi k}\frac{S_{0}}{\left(d_{0}+\Delta d\right)}\right|=\left|\left(C_{1}+\Delta C_{1}\right)-\left(C_{2}-\Delta C_{2}\right)\right|=\left|\Delta C_{1}+\Delta C_{2}\right|$$

Therefore, the carriers’ acceleration can be precisely measured with the capacitance variation. Moreover, the stiffness of sensing circuit and driving circuit determines MEMS accelerometer’s accuracy. Owing to the fact that their stiffness is related to Si-based material’s stiffness, ambient temperature as the most important factor to Si-based material’s stiffness is fundamental [18]. Sensing circuit and driving circuit deform as ambient temperature varies, and especially some errors appear in the capacitance of the sensing circuit, which is the corresponding TDE ∆*E_MEMS_* in MEMS accelerometer’s output. Hence, the conventional TDE estimation model introduces a microstructure linear analytical method in MEMS accelerometer’s deformation. Considering that Si-based material’ deformation stays with ambient temperature variation ∆*T* and its square ∆*T*^2^, the conventional TDE estimation model is shown:

(4) $$\Delta E_{MEMS}=f\left(\Delta T,\Delta T^{2}\right)$$

#### 2.1.2. Microstructure Analysis of Si-Based Material under Temperature Variation

**Figure 2:**
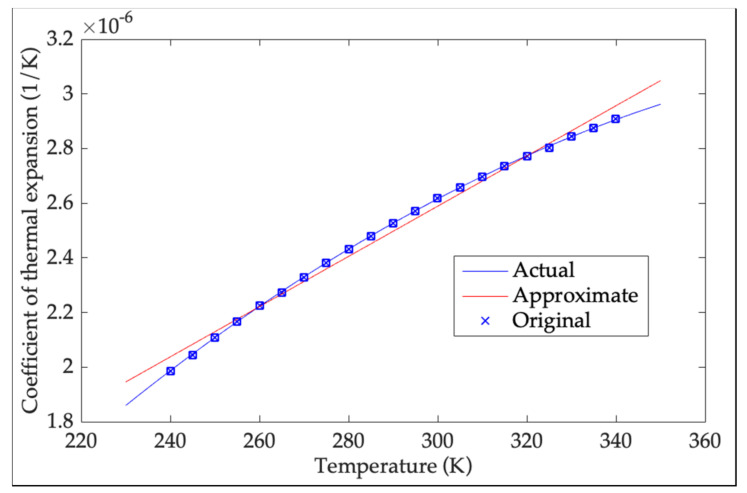
Comparison of CTE variation in actual and approximate situations.

The conventional TDE estimation model is built with microstructure linear analytical method, and its structural sizes deform linearly in 3 dimensions using constant CTE. However, CTE of Si-based material varies nonlinearly as temperature variation, which causes its structure to deform nonlinearly in large temperature range and brings nonlinear variation in the distance between crystal lattice structures of Si-based material. Therefore, it is necessary to apply microstructure nonlinear analytical method in CTE of Si-based material to modify the conventional model and estimate more accurate TDE. Figure 2 shows the comparison between the actual CTE of Si-based material and its approximate one [25].

**Figure 3:**
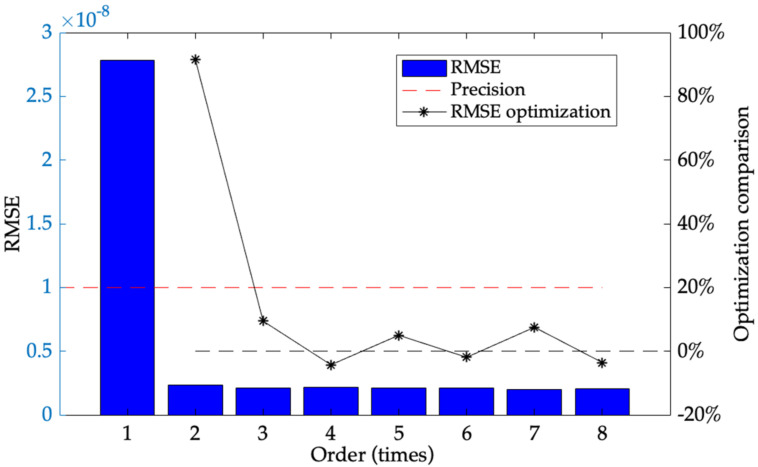
The comparison of RMSE in each order.

Figure 2 shows the prominent deviation between the actual CTE of Si-based material and its approximate one, which means there are currently some errors in TEP of Si-based material analyzed and utilized. Under the condition, TDE traceability is inevitably inaccurate and never obtains the accurate TRQ, and a precise TDE estimation model cannot be built. Therefore, it is helpful to comprehensively analyze TEP of Si-based material. According to linear TEP of Si-based material, all the sizes inside MEMS accelerometer have linear variation as ambient temperature. Taking a size *l*_0_ at *T*_0_ as an example, from Thermal Expansion Formula its size changes to *l*_1_ at *T*_1_, which is shown as follows:

(5) $$l_{1}=l_{0}\left(\Delta T\alpha +1\right)$$

where, *α* is constant CTE of Si-based material, ∆*Tα* is its TEP. According to Figure 2, TEP of Si-based material shows nonlinearity as ambient temperature. A prerequisite to show size variation as ambient temperature is accurately obtaining CTE of Si-based material, and it is essential to use a suitable method to describe the nonlinearity. Polynomial fitting is classical in curve fitting and can obtain accurate results with a small amount of test data [26]. It presents all kinds of a priori relationships with easy implementation, nonlinearity and linearity [27]. According to Figure 2, the relationship between CTE and ambient temperature is a significant simple nonlinear one, which shows that polynomial fitting is a suitable method to describe accurate CTE of Si-based material. Its order is crucial to determine its accuracy. The lower order causes some remarkable fitting errors, but it is easy to implement. Although higher order increases fitting accuracy, it introduces excessive complexity which brings slow fitting convergence and bad calculation in real time. To determine proper fitting order, Root Mean Square Error (RMSE) is a criterion to show its fitting performance. The smaller RMSE is, the more accurately polynomial fitting describes the targeted relationship. When RMSE cannot be higher as fitting order increases, current fitting order is optimal and the higher one is helpless except harming its real time. Ideally, fitting error is less than 10% of the accuracy of the targeted relationship. CTE of Si-based material is 2.5 × 10^−6^/K with the ac curacy of 1 × 10^−7^/K at 323 K, and its RMSE should be less than 1 × 10^−8^*/K* [25]. From Reference [28], the relationship between TEP of Si-based material and CTE in 240–340 K is described by polynomial fitting, and Figure 3 shows RMSE of each order.

From Figure 3, RMSE of 2-order has been improved to 10% of that of 1-order, and RMSE of 2-order fitting is 2.39 × 10^−9^/K, less than 1 × 10^−8^/K. Although RMSE of 3-order and higher order is less than 1 × 10^−8^/K, their RMSE cannot be reduced further remarkably as the fitting order increases, which demonstrates that ambient temperature *T* and its square *T*^2^ are the only decisive factors to CTE. Therefore, 2-order fitting is used in CTE of Si-based material *α*(*T*) at ambient temperature *T*, and its estimation model is obtained as follows:

(6) $$\alpha \left(T\right)=-5.429\times 10^{-6}+2.79\times 10^{-8}T-3.226\times 10^{-11}T^{2}$$

#### 2.1.3. Modification of the Conventional TDE Precise Estimation Model

Assuming that the input of MEMS accelerometer is constant, its output remains constant as *a*_0_ at ambient temperature *T*_0_. Due to its structural deformation, its output at *T* is *a*(*T*) which includes the truth value *a*_0_ and TDE ∆*a*(*T*). Then, its output is shown as follows:

(7) $$a\left(T\right)=a_{0}+\Delta a\left(T\right)$$

From MEMS accelerometer’s principle, *a*_0_ is related to its capacitance variation under ideal conditions ∆*C*_0_, *a*_0_∝∆*C*_0_. When ambient temperature varies, its structural deformation induces its capacitance variation, *a*(*T*)∝∆*C*(*T*). Then, ∆*a*(*T*) can be deduced further:

(8) $$\Delta a\left(T\right)=\left[a_{0}-a\left(T\right)\right]\propto \left[\Delta C_{0}-\Delta C\left(T\right)\right]$$

**Figure 4:**
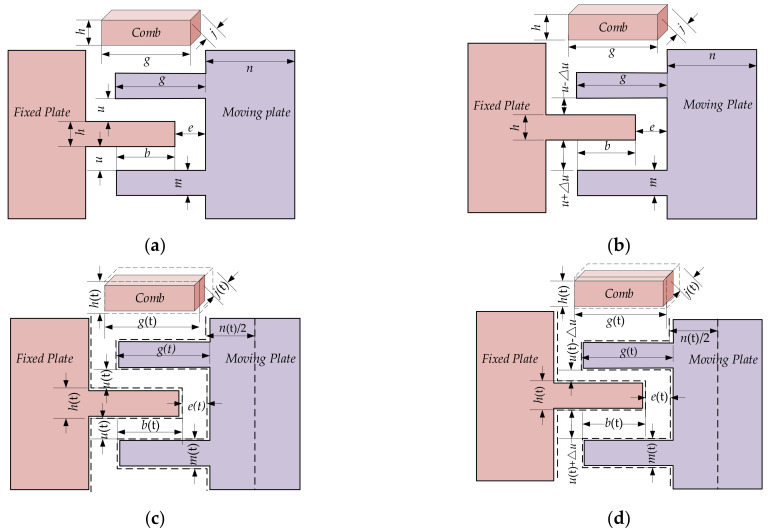
Structural deformation of MEMS accelerometer in diverse conditions. (**a**) Local structure under static state without TEP. (**b**) Local structure under working state without TEP. (**c**) Local structure under static state with TEP. (**d**) Local structure under working state with TEP.

According to Figure 1, mass, driving circuit, and sensing circuit are assembled on substrate. Further, the substrate deforms as ambient temperature, mass, driving circuit, and sensing circuit displace together, and the relative distances between them remain stable, especially the combs. Given that MEMS accelerometer’s output is related to the capacitance between the combs, substrate deformation has no impact on MEMS accelerometer’s output at all, and it is a negligible factor to MEMS accelerometer’s accuracy. Then, the influence of ambient temperature on the capacitance between combs is analyzed using CTE estimation model in diverse conditions, shown in Figure 4.

Here, *b* is overlap length between moving plate and fixed plate, *e* is the distance between comb of fixed plate and long beam of moving plate, *h* is comb width of fixed plate, *g* is comb length, *u* is comb distance between moving plate and fixed plate, *m* is comb width of moving plate, *n* is the width of long beam of moving plate, *j* is the thickness of comb. Therefore, the capacitance between the combs *Cs* under static state without TEP is shown:

(9) $$C_{S}=C_{1}+C_{2}+C_{3}$$

where, *C*_1_ is the capacitance between the short side of comb of fixed plate and long beam of fixed plate, *C*_2_ is the capacitance between the upper edge of comb of fixed plate and the lower edge of comb of moving plate, *C*_3_ is the capacitance between the lower side of comb of fixed plate and the upper side of comb of moving plate. Therefore, *C*_1_, *C*_2_, and *C*_3_ can be defined:

(10) $$\begin{array}{cc} C_{1}=\frac{\varepsilon S}{d_{1}}=\frac{\varepsilon hj}{e} & C_{2}=C_{3}=\frac{\varepsilon bj}{u} \end{array}$$

where, *ε* is dielectric constant of Si-based material. Substituting (10) into (9), *C_s_* is shown:

(11) $$C_{s}=C_{1}+C_{2}+C_{3}=\frac{\varepsilon hj}{e}+2\frac{\varepsilon bj}{u}$$

According to Figure 4b, when the carriers accelerate to *A*_0_, a displacement occurs between the upper and lower combs, and *C*_1_′, *C*_2_′ and *C*_3_′ can be described as follows:

(12) $$\begin{array}{ccc} {C^{\prime }}_{1}=\frac{\varepsilon hj}{e} & {C^{\prime }}_{2}=\frac{\varepsilon bj}{u-\Delta u} & {C^{\prime }}_{3}=\frac{\varepsilon bj}{u+\Delta u} \end{array}$$

Then, the capacitance under working state without TEP *C_w_* is shown:

(13) $$C_{w}={C^{\prime }}_{1}+{C^{\prime }}_{2}+{C^{\prime }}_{3}=\frac{\varepsilon hj}{e}+\frac{\varepsilon bj}{u-\Delta u}+\frac{\varepsilon bj}{u+\Delta u}$$

According to (11) and (13), the capacitance variation ∆*C* can be expressed:

(14) $$\Delta C=\left|C_{s}-C_{w}\right|=\left|\frac{\varepsilon bju}{u-\Delta u}+\frac{\varepsilon bju}{u+\Delta u}-2\frac{\varepsilon bj}{u}\right|$$

However, MEMS accelerometer’s internal structure changes nonlinearly as CTE of Si-based material. Using CTE estimation model of Si-based material, Figure 4c shows local structure of MEMS accelerometer under static state with TEP. Due to its symmetrical structure, the actual deformation of long beam of moving plate to the fixed plate is half of its dimensional variation. From (5), its structure varies as ambient temperature and is expressed:

(15) $$\begin{array}{cccc} \begin{array}{c} h\left(T\right)=h\left[1+\alpha \left(T\right)\right]\\ m\left(T\right)=m\left[1+\alpha \left(T\right)\right] \end{array} & \begin{array}{c} j\left(T\right)=j\left[1+\alpha \left(T\right)\right]\\ b\left(T\right)=b+\alpha \left(T\right)k_{1} \end{array} & \begin{array}{c} g\left(T\right)=g\left[1+\alpha \left(T\right)\right]\\ u\left(T\right)=u-\alpha \left(T\right)k_{2} \end{array} & \begin{array}{c} n\left(T\right)=n\left[1+\alpha \left(T\right)\right]\\ e\left(T\right)=e-\alpha \left(T\right)k_{3} \end{array} \end{array}$$

where, *k*_1_ *= i + n/*2 *+ g*, *k*_2_ *= (m + k)/*2, *k*_3_ *= g + n/*2. From (10), *C*_1_ at temperature *T C*_1_(*T*), *C*_2_ at temperature *T C*_2_(*T*) and *C*_1_ at temperature *T C*_3_(*T*) can be deduced as follows:

(16) $$\begin{array}{cc} C_{1}\left(T\right)=\frac{\varepsilon hj{\left[1+\alpha \left(T\right)\right]}^{2}}{e-\alpha \left(T\right)k_{3}} & C_{2}\left(T\right)=C_{3}\left(T\right)=\frac{\varepsilon j\left[b+\alpha \left(T\right)k_{1}\right]\left[1+\alpha \left(T\right)\right]}{u-\alpha \left(T\right)k_{2}} \end{array}$$

By substituting (15) into (16), *C_s_* at temperature *T C_s_*(*T*) is further shown as follows:

(17) $$C_{s}\left(T\right)=C_{1}\left(T\right)+C_{2}\left(T\right)+C_{3}\left(T\right)=\frac{\varepsilon hj{\left[1+\alpha \left(T\right)\right]}^{2}}{e-\alpha \left(T\right)k_{3}}+\frac{2\varepsilon j\left[b+\alpha \left(T\right)k_{1}\right]\left[1+\alpha \left(T\right)\right]}{u-\alpha \left(T\right)k_{2}}$$

Figure 4d shows local structure variation of MEMS accelerometer under working state with TEP. When ambient temperature varies to *T*, *C*_1_′(*T*), *C*_2_′(*T*) and *C*_3_′(*T*) are expressed:

(18) $$\begin{array}{ccc} {C^{\prime }}_{1}\left(T\right)=\frac{\varepsilon hj{\left[1+\alpha \left(T\right)\right]}^{2}}{e-\alpha \left(T\right)k_{3}} & {C^{\prime }}_{2}\left(T\right)=\frac{\varepsilon j\left[b+\alpha \left(T\right)k_{1}\right]\left[1+\alpha \left(T\right)\right]}{u-\alpha \left(T\right)k_{2}-\Delta u} & {C^{\prime }}_{3}\left(T\right)=\frac{\varepsilon j\left[b+\alpha \left(T\right)k_{1}\right]\left[1+\alpha \left(T\right)\right]}{u-\alpha \left(T\right)k_{2}+\Delta u} \end{array}$$

From (13), the capacitance under working state with TEP *C_w_(T)* can be shown:

(19) $$\begin{array}{c} C_{w}\left(T\right)={C^{\prime }}_{1}\left(T\right)+{C^{\prime }}_{2}\left(T\right)+{C^{\prime }}_{3}\left(T\right)\\ =\frac{\varepsilon hj{\left[1+\alpha \left(T\right)\right]}^{2}}{e-\alpha \left(T\right)k_{3}}+\frac{\varepsilon j\left[b+\alpha \left(T\right)k_{1}\right]\left[1+\alpha \left(T\right)\right]}{u-\alpha \left(T\right)k_{2}-\Delta u}+\frac{\varepsilon j\left[b+\alpha \left(T\right)k_{1}\right]\left[1+\alpha \left(T\right)\right]}{u-\alpha \left(T\right)k_{2}+\Delta u} \end{array}$$

Therefore, the capacitance variation ∆*C*(*T*) at temperature *T* can be shown as follows:

(20) $$\begin{array}{l} \Delta C\left(T\right)=\left|C_{s}\left(T\right)-C_{w}\left(T\right)\right|\\ \;\;\;=\left|\frac{\varepsilon j\left[b+\alpha \left(T\right)k_{1}\right]\left[1+\alpha \left(T\right)\right]}{u-\alpha \left(T\right)k_{2}-\Delta u}+\frac{\varepsilon j\left[b+\alpha \left(T\right)k_{1}\right]\left[1+\alpha \left(T\right)\right]}{u-\alpha \left(T\right)k_{2}+\Delta u}-\frac{2\varepsilon j\left[b+\alpha \left(T\right)k_{1}\right]\left[1+\alpha \left(T\right)\right]}{u-\alpha \left(T\right)k_{2}}\right| \end{array}$$

Then, TDE of MEMS accelerometer ∆C-∆C(*T*) can be shown as follows:

(21) $$\begin{array}{l} \Delta C-\Delta C\left(T\right)=\left|\frac{\varepsilon bju}{u-\Delta u}+\frac{\varepsilon bju}{u+\Delta u}-2\frac{\varepsilon bj}{u}\right|\\ -\left|\frac{\varepsilon j\left[b+\alpha \left(T\right)k_{1}\right]\left[1+\alpha \left(T\right)\right]}{u-\alpha \left(T\right)k_{2}-\Delta u}+\frac{\varepsilon j\left[b+\alpha (T)k_{1}\right]\left[1+\alpha \left(T\right)\right]}{u-\alpha \left(T\right)k_{2}+\Delta u}-\frac{2\varepsilon j\left[b+\alpha (T)k_{1}\right]\left[1+\alpha \left(T\right)\right]}{u-\alpha \left(T\right)k_{2}}\right| \end{array}$$

According to (21), it concludes that there is a deviation when ambient temperature varies, which is related to *α*(*T*) and *α*(*T*)^2^. From (6), *α*(*T*) is related to *T* and *T*^2^, which deduces the deviation is also related to *T* and *T*^2^. Hence, the conventional model can be modified and a novel TDE precise estimation model for MEMS accelerometer is established:

(22) $$\Delta E_{MEMS}=f\left(\Delta T,\Delta T^{2},T,T^{2}\right)$$

### 2.2. Parameter Identification Optimization for Novel TDE Precise Estimation Model

After a novel TDE precise estimation model for MEMS accelerometer is established, its parameter identification optimization determines TDE estimation accuracy. The more accurate it is, the more precisely TDE is estimated. Therefore, the prerequisite to its implementation is testing TDE accurately, and a proper TDE test method is necessary.

#### 2.2.1. TDE Test Method

TDE of MEMS accelerometer is described with bias and scale factor as well as random error. Once it is manufactured, the physical characteristics are fixed. The presence of bias, scale factor, and random error causes different MEMS accelerometers to have diverse environmental adaptability. According to the datasheets, their TDE can be grossly reckoned:

(23) ΔE=αΔT+βΔT

where, ∆*E* are roughly reckoned values of TDE, *α* is its character “Zero-rate level change vs. temperature”, *β* is its character “Sensitivity change vs. temperature” [18]. Theoretically, ∆*E* is slightly smaller than TDE ∆*E_MEMS_*, so we can obtain:

(24) $$\Delta E\leq \Delta E_{MEMS}$$

MEMS accelerometer’s sensitivity ∆*E_S_* determines its measured minimum. When ambient temperature deteriorates during it works, it is possible TDE is much greater than ∆*E_S_*, even completely submerging to worsen MEMS accelerometer’s output. Therefore, it is essential for ambient temperature to vary slowly, which avoids some large accidental output deviations to ensure its accuracy. To test TDE precisely, temperature control interval is shown:

(25) $$\Delta E_{S}\approx \Delta E_{MEMS}$$

Besides, to ensure the accuracy and the real time of the test results, some other important factors should be considered, which are shown as follows:

1. Heat conduction measures

Heat conduction measures should be taken to reduce heat conduction delay, which keeps ambient temperature the same as the MEMS accelerometer.

2. Precise temperature measurement system

Precise temperature measurement system is essential to precisely measure ambient temperature. It needs be installed closely on MEMS accelerometer, and its measurement accuracy needs to be 2-times more precise than ambient temperature variation. Further, its measurement frequency should be higher than MEMS accelerometer’s output frequency to record test results as real time as much as possible [18].

3. Reasonable temperature control period

**Figure 5:**
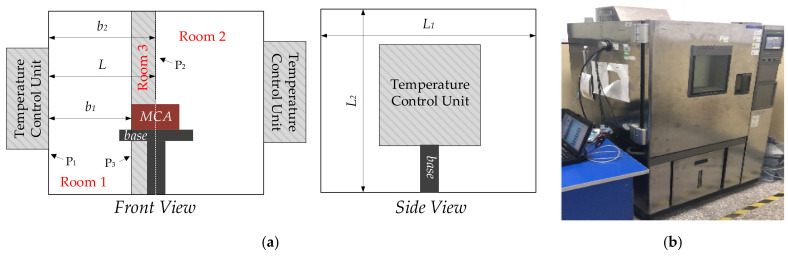
The schematic diagram of test platform. (**a**) The installation of MEMS accelerometer inside the thermal chamber; (**b**) field test of TDE test method.

From heat conduction theory, it takes some time to transfer heat from point A to point B. Sufficient temperature control period makes temperature variation at point A the same as point B, but an insufficient one causes temperature at point B not to stabilize at required temperature; in that case TDE cannot be tested precisely. Usually, thermal chamber is used to test MEMS accelerometer, and it integrates temperature control system with temperature control units on both sides. Further, it adopts a structure design of front-door open and closed insulation to ensure its control effectiveness. It takes time for heat transferring determined by heat conducting condition. Better heat conducting condition requires less time to maintain temperature, and temperature variation inside is truly reflected. Therefore, temperature sensors are set close to MEMS accelerometer, and temperature control units are set in a distance to transfer heat uniformly. Figure 5 shows a schematic diagram of the test platform.

From Figure 5, temperature control units control ambient temperature in the thermal chamber through its inner wall. Taking the center of MEMS accelerometer installed at the center of the thermal chamber as a reference, the thermal chamber is divided into two complete identical rooms (Room 1 and Room 2) using the plane *P*_1_ with its inner wall parallel to the center of thermal chamber. Assuming that the sizes of two rooms are *L × L*_1_ *× L*_2_, heat from temperature control units uniformly transfers to their joint plane *P*_2_ along length *L* perpendicular to the inner wall. The farther the location is away from the inner wall, the longer heat transfers. The joint plane is the last area where ambient temperature stabilizes. According to Fourier’s law, conduction heat transfer equation is shown:

(26) $$w_{b}=-\lambda A\frac{dt}{dx}$$

where, *w_b_* is heat conduction rate of any point B in Room 1 or Room 2, *A* is the area of the joint plane, *dt*/*dx* is temperature gradient, *λ* is coefficient of heat conductivity. By deforming (26) and integrating, we obtain (27) as follows:

(27) $$\begin{array}{cc} \int_{0}^{b}w_{b}dx=\int_{T_{0}}^{T_{b}}-\lambda AdT & w_{b}=\frac{-\lambda A\left(T_{b}-T_{0}\right)}{b} \end{array}$$

where, *b* is the vertical distance from any point B to the inner wall in Room 1 or Room 2, *T_b_* is ambient temperature at point B, *T*_0_ is ambient temperature of the inner wall. According to (27), when temperature varies from *T*_0_ to *T_b_*, its required power at point B can be directly calculated. Therefore, assuming that the heating period is *t*_s_, its required energy *Q_b_* at point B is shown as follows:

(28) $$Q_{b}=\frac{\lambda A\left|T_{b}-T_{0}\right|}{b}t_{s}$$

From Figure 5, MEMS accelerometer is installed perpendicular to the inner wall. The plane *P*_3_ parallel to the inner wall where its left endpoint stays in and *P*_2_ as well as the thermal chamber form Room 3. To test TDE accurately, Room 3 should be heated evenly and completely. From (28), it needs different energy at any point of Room 3 with different temperature variation. To obtain the overall energy to heat Room 3, (28) is integrated and shown:

(29) $$\begin{array}{cc} \int_{b_{1}}^{b_{2}}Q_{b}db=\int_{b_{1}}^{b_{2}}\frac{\lambda A\left|T_{b}-T_{0}\right|t_{s}}{b}db & Q=\lambda A\left|T_{b}-T_{0}\right|t_{s}\left|\ln\left(b_{2}\right)-\ln\left(b_{1}\right)\right| \end{array}$$

where, *b*_1_ is the vertical distance between *P*_3_ and *P*_1_, *b*_2_ is the vertical distance between *P*_2_ and *P*_1_. According to specific heat capacity formula, the heat heating Room 3 entirely and uniformly can be expressed as follows:

(30) $$Q=Cm\Delta T=C\Delta TA\left(b_{2}-b_{1}\right)$$

where, *C* is specific heat capacity of air in Room 3 in a closed condition, *m* is its mass, ∆*T* is temperature variation and ∆*T = |T_b_-T*_0_*|*. Based on (29) and (30), a new equation is obtained:

(31) $$t_{s}=\frac{C\Delta TA\left(b_{2}-b_{1}\right)\rho }{\lambda A\left|T_{b}-T_{0}\right|\left|\ln\left(b_{2}\right)-\ln\left(b_{1}\right)\right|}=\frac{C\left(b_{2}-b_{1}\right)\rho }{\lambda \left|\ln\left(b_{2}\right)-\ln\left(b_{1}\right)\right|}$$

where, *ρ* is air density inside thermal chamber. From (31), the time for heat conduction from the inner wall to the joint of two rooms can be calculated. To ensure the thermal chamber is heated uniformly, temperature control period *t_p_* is shown as follows:

(32) $$t_{s}\leq t_{p}$$

Based on (25) and (32), MEMS accelerometer IIS328DQ is chosen randomly to test its TDE. According to its datasheet, ∆*E_S_* = 0.98 mg/digit, *α* = ±0.8 mg/°C, and its maximum operating temperature range is −40 °C~85 °C. After dimensional transformation, *β* is obtained:

(33) $$\beta =\frac{0.02\%\times FS}{\left(85\;{}^{\circ}C\right)-\left(-40\;{}^{\circ}C\right)}=\frac{0.02\%\times 4\;g}{125}=0.64\;\mathrm{mg}/{}^{\circ}C$$

According to (25), temperature control interval ∆*T* can be shown as follows:

(34) $$\Delta T\leq \frac{0.98\;\mathrm{mg}/\mathrm{digit}}{\left[\left(+0.8\;\mathrm{mg}/{}^{\circ}C\right)-\left(-0.8\;\mathrm{mg}/{}^{\circ}C\right)\right]+0.64\;\mathrm{mg}/{}^{\circ}C}\approx 0.43\;{}^{\circ}C$$

To simplify test steps, ∆*T* = 0.4 °C. Thermal chamber SET-Z-021 is used to test IIS328DQ, and its parameters *C =* 1.005 kJ/(kg × K), *λ =* 0.0267 W/m °C, *L =* 0.6 m, *ρ =* 1.293 kg/m^3^. We then obtain:

(35) $$t_{s}=29.323\;s$$

From (35), temperature control period 29.232 s is taken for temperature control units to vary temperature control interval 0.4 °C. To simplify test steps and reserve an allowance for stable temperature transfer, *t_p_* = 35 s. IIS328DQ is tested and its temperature is obtained using precise temperature measurement system with accuracy of ±0.03 °C and frequency of 10 Hz [18]. Therefore, the temperature experiment is designed and shown as follows:

1. IIS328DQ is installed on the base of thermal chamber, its measuring direction is vertically down and its true value 1 g. Temperature sensors for the precise temperature measurement system are attached on it. Wireless transmission module sends test results and PC is prepared to receive its temperature *T_a_* and its output *D_a_*.
2. Cool thermal chamber to lower limit of the rated operating temperature range −20 °C, and keep *T_a_* and *D_a_* recording for 1 h after ambient temperature stays stable.
3. Heat thermal chamber to higher limit of the rated operating temperature range 50 °C at a rate of 41 °C/h, which is 0.4 °C per 35 s. When *T_a_* goes to 50 °C, stop the test until it stays stable for an hour.
4. Repeat step (2) to (3) three times, and choose one of them randomly as the test results.

**Figure 6:**
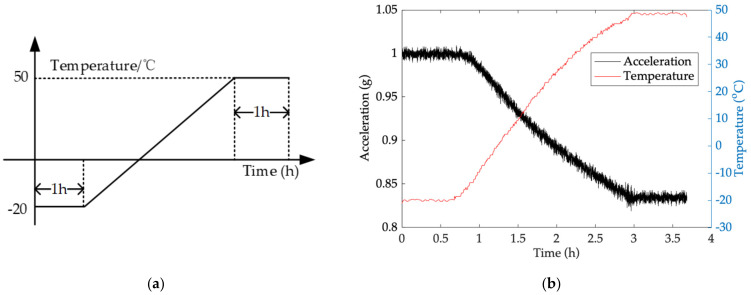
Flow chart of temperature experiment and the test results. (**a**) Test flow; (**b**) the test results.

Figure 6 shows the flow chart of temperature experiment and the test results.

#### 2.2.2. Implementation of Novel Model Based on PSO-GA-BPNN

After accurately testing TDE and TCQ, another key factor for TDE precise estimation is establishing a suitable model with accurate parameters. Considering there may be complex nonlinear relationships between TCQ and TDE, it is essential to apply a comprehensive model. BPNN with nonlinear structure can describe linearity and nonlinearity under arbitrary precision. Based on a number of test results, its parameters are precisely identified and its structure is built. Therefore, BPNN can be used in TDE estimation. Then, the conventional TDE estimation model for MEMS accelerometer and the novel one are shown:

(36) $$\begin{array}{cc} \Delta E_{MEMS}^{1}=f_{BPNN}\left(\Delta T,\Delta T^{2}\right) & \Delta E_{MEMS}^{2}=f_{BPNN}\left(\Delta T,\Delta T^{2},T,T^{2}\right) \end{array}$$

**Figure 7:**
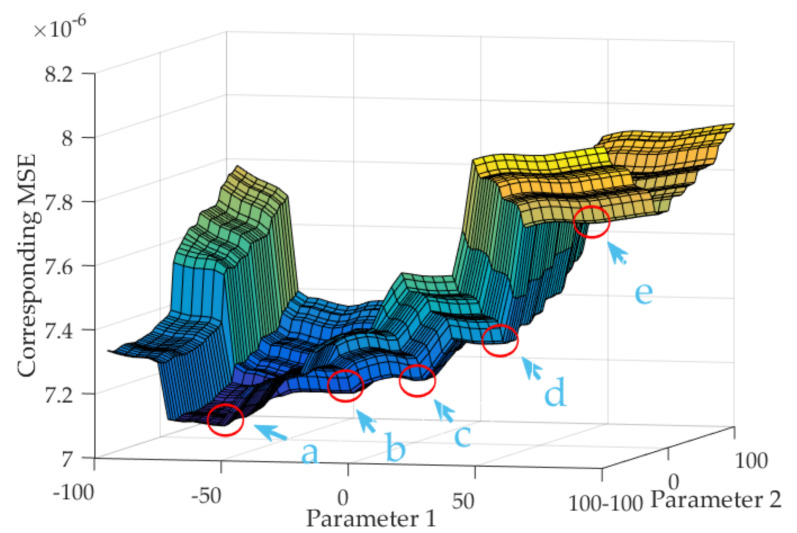
The local optimums of BPNN in some ranges.

After being trained and implemented, the novel TDE estimation model and its outputs are stably established. If its structure and parameters change, its performance changes as well. However, BPNN has local optimums in some ranges; global optimums may inevitably degenerate into non-global optimums in the whole range, shown in Figure 7.

As shown in Figure 7, several concave surfaces appear as its parameters change. When the parameters change close to the concave surface in gradient operation, although BPNN outputs stably, they are the local optimums. Even so, there may be multiple concave regions and many local optimums. Therefore, eliminating local optimums is an important way to obtain an accurate BPNN. Assuming *X* is the positions of weights *ω* and thresholds *b* of BPNN in solution space of cost function, its objective function can be expressed as *Y = f*(*X*). When *Y* is unequal to its expected value $\bar{Y}$, its cost function of searching *X* can be expressed:

(37) $$E\left(X\right)=\frac{1}{2}{\left(Y-\bar{Y}\right)}^{2}$$

The current position *x* of *X* in the solution space is obtained by the position update operator, which is shown as follows:

(38) $$x=x_{0}-\sum_{k=1}^{m}\eta \frac{dE\left(x_{k}\right)}{dx_{k}}$$

where, *k* is the number of searching (*k* < *m*), *η* is learning rate, *x*_0_ is the initial position of *X*. As shown in Figure 7, when *x*_0_ is in *d*-region, the solution of *X* can be obtained with (38), and *x_d_* is its local optimum. When *x*_0_ locates in *a*-region, the solution of *X* is its global optimal solution. Therefore, the initial position of *x*_0_ is critical to obtain the global optimums. Then, GA is introduced to optimally choose the initial position *x*_0_ of *X*. Its core operation is crossover and mutation, which are carried out by simulating biological chromosome genes in natural evolution process. Therefore, the global optimal solution of BPNN is transformed into genes with the best fitness [29]. GA adopts the binary encode method, and each binary part represents a gene. The binary code of *ω* and *b* are substituted into the initial position *x*_0_ as follows:

(39) $${\left(x_{0}\right)}_{2}={\left[\ldots \left(a_{j0}\ldots a_{jN_{1}}\right)\ldots \left(a_{i0}\ldots a_{iN_{1}}\right)\ldots \right]}_{2}$$

where, *N*_1_ is the binary digit number of binary encoding with *ω* and *b*. Crossover operator of GA obtains the gene of filial generation *x_c_*, and mutation operator selects one or more loci in *x_c_* randomly, and gene values of these loci mutate to ${x^{\prime }}_{ci}$. *ω* and *b* of the modified BPNN with GA obtained by decoding individual ${x^{\prime }}_{ci}$ and substituting into BPNN. The fitness of an individual ${x^{\prime }}_{ci}$ in the population is calculated by the following fitness function:

(40) $$f\left({x^{\prime }}_{ci}\right)=\left|y\left({x^{\prime }}_{ci}\right)-\bar{y}\left({x^{\prime }}_{ci}\right)\right|$$

Through more crossover and mutation, the individual *x_old_* with high fitness is selected and the expected initial solution position of BPNN *x_new_* is obtained as follows:

(41) $$x_{new}=x_{old}+x_{c}$$

when *x_new_* locates to *a*-region, *b*-region and *c*-region; the approximation from *x_new_* to the global optimal solution *x_a_* is operated by the mutation. Then, the novel TDE estimation model based on GA-BPNN is expressed further as follows:

(42) $$\Delta E_{MEMS}^{3}=f_{GA-BPNN}\left(\Delta T,\Delta T^{2},T,T^{2}\right)$$

However, the mutation process of GA is probabilistically disordered and needs more iterations in a flat region, which shows GA has poor calculation real time to maintain its perfect local optimization ability. PSO has solving directivity to reduce complex iterations and rapidly converge to the targets, which can assist GA with less iterations to get the global optimum. Therefore, PSO is used in GA-BPNN to enhance its calculation real time. Using the individual information shared in group, the iteration evolves from disorder to order in the solution space of GA, and the process of solving *x_a_* with *x_new_* is similar to that of bird-flock foraging behavior [30]. After crossover and mutation, PSO selects *x_new_* from GA as its initial position *x*_0_, which is obtained by velocity update operator and position operator, expressed as:

(43) $$x_{0}=\sum_{i=1}^{n}\left[v_{i}+c_{1}r_{1}\left(p_{i}-x_{i}\right)+c_{2}r_{2}\left(g_{i}-x_{i}\right)\right]$$

where, *v_i_* is particle velocity, *r*_1_ and *r*_2_ are random values during 0~1, *c*_1_ and *c*_2_ are constant, *p*_i_ is historical optimum of particle, *g_i_* is historical optimum of particle swarm, *n* is iteration number of PSO. By substituting (38) into (43), cost function solution *x* of BPNN is shown:

(44) $$x=\sum_{i=1}^{n}\left[v_{i}+c_{1}r_{1}\left(p_{i}-x_{i}\right)+c_{2}r_{2}\left(g_{i}-x_{i}\right)\right]-\sum_{j=1}^{m}\eta \frac{dE\left(x_{j}\right)}{dx_{j}}$$

**Figure 8:**
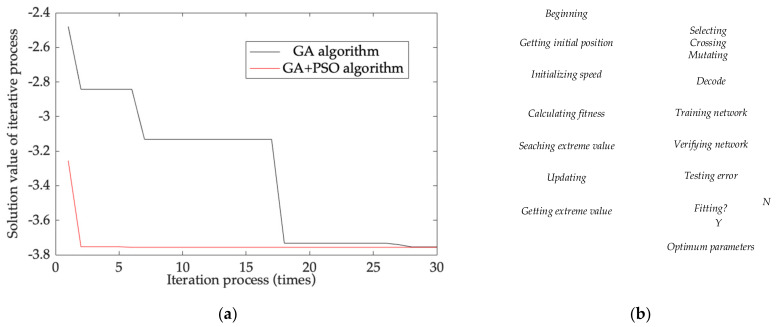
Performance improvement and its implementation. (**a**) Performance improvement comparison of GA and PSO-GA; (**b**) implementation flow chart of the novel model.

BPNN is trained with mathematical calculation software, such as Mathematica or Python. Complete TCQ are used as its inputs and TDE as its output, and BPNN is built after being trained. GA and PSO-GA are also implemented in BPNN with codes. BPNN is optimized in two ways, training samples’ initial value and cost function solution. The first one optimizes the center vector and the width of Gauss function as the kernel functions of the neurons in hidden layer, and the second one optimizes the weights between the neurons in output and hidden layers. To verify the optimization performance of PSO on GA, GA and PSO-GA are compared with performance evaluation equation, which is shown below:

(45) $$z=-y\sin\left(2\pi x\right)-x\cos\left(2\pi y\right)$$

where, *x*∈[−2, 2], *y*∈[−2, 2]. The smaller *z* is, the fewer iterations PSO on GA have. Figure 8 shows performance improvement and its implementation.

From Figure 8, GA approaches the global optimum after 27 iterations and has the same iterations as PSO-GA with the same TDE estimation accuracy. By comparison, PSO-GA does that after 3 iterations, and the iterations are reduced by 88.9%. Therefore, after introducing PSO into GA, the calculation real time of GA is significantly improved, which guarantees that the TDE of the MEMS accelerometer can be estimated in a more accurate and timely manner. Then, the novel TDE estimation model based on PSO-GA-BPNN is shown as follows:

(46) $$\Delta E_{MEMS}=f_{PSO-GA-BPNN}\left(T,T^{2},\Delta T,\Delta T^{2}\right)$$

## 3. Experiments and Analysis

**Figure 9:**
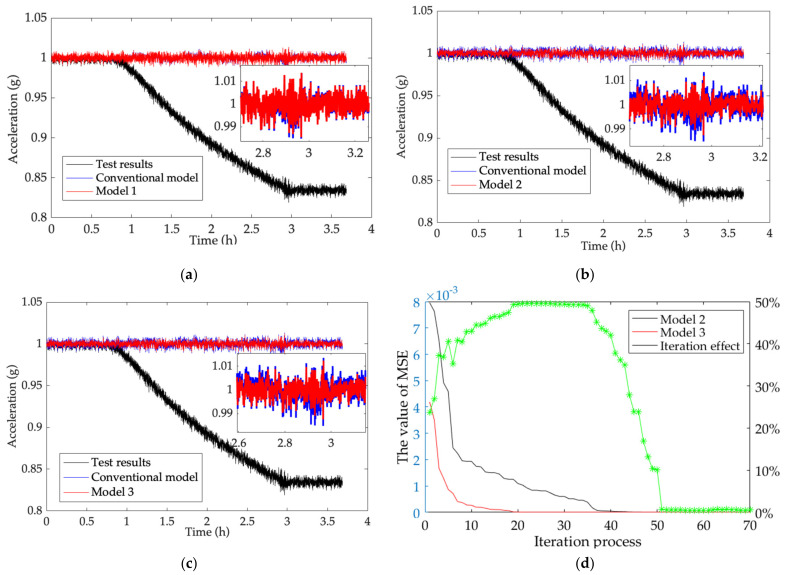
Comparison of performance improvement between the novel and the conventional models. (**a**) Accuracy improvement of Model 1; (**b**) accuracy improvement of Model 2; (**c**) accuracy improvement of Model 3; (**d**) real-time improvement of Model 2 and Model 3.

In order to estimate the TDE estimation performance of the novel TDE estimation model for the MEMS accelerometer, one group of the test results is chosen to ensure test universality. From (36), (42) and (46), the conventional model based on TCQ (∆*T*, ∆*T*^2^) and BPNN as well as the novel model are established, and their performances on accuracy and real time are compared. Figure 9 shows performance improvements in TDE estimation accuracy of the novel model based on TCQ (*T*, *T*^2^, ∆*T*, ∆*T*^2^) and BPNN (Model 1), the novel model based on TCQ (*T*, *T*^2^, ∆*T*, ∆*T*^2^) and GA-BPNN (Model 2), the novel model based on TCQ (*T*, *T*^2^, ∆*T*, ∆*T*^2^) and PSO-GA-BPNN (Model 3), and performance improvement in TDE estimation real time between Model 2 and Model 3.

From Figure 6, ambient temperature at −20 °C is considered as a reference, and it gradually increases to 50 °C and stays stable. TDE are estimated by Model 1, Model 2 and Model 3 and the test results are compensated well. The output of the MEMS accelerometer fluctuates around its true value (1 g) and its TDE is reduced greatly by introducing GA, which decouples the MEMS accelerometer from ambient temperature to enhance its environmental adaptability. Besides, the test results demonstrate that TCQ has no excitation to TDE and no influence on its output accuracy, and the novel model has perfect TDE estimation to ensure that the MEMS accelerometer has better performance. To show the performance improvement in TDE estimation, the performance is evaluated with the following equation:

(47) $$\begin{array}{ccc} Q_{1}=\frac{\left|MSE_{Model1}-MSE_{CM}\right|}{MSE_{CM}}\times 100\% & Q_{2}=\frac{\left|MSE_{Model2}-MSE_{CM}\right|}{MSE_{CM}}\times 100\% & Q_{3}=\frac{\left|MSE_{Model3}-MSE_{CM}\right|}{MSE_{CM}}\times 100\% \end{array}$$

where, *Q*_1_ is the performance improvement of Model 1, *Q*_2_ is the performance improvement of Model 2, *Q*_3_ is the performance improvement of Model 3; *MSE_CM_* is MSE of the test results compensated by the conventional model, *MSE_Model_*_1_ is MSE of the test results compensated by Model 1, *MSE_Model_*_2_ is MSE of the test results compensated by Model 2, *MSE_Model_*_3_ is MSE of the test results compensated by Model 3. Then, to comprehensively demonstrate TDE estimation performance improvements for Model 1, Model 2 and Model 3, Table 1 shows all performance of the test results in three tests and its improvement.

According to Table 1, compared with the conventional model, the outputs of the MEMS accelerometer compensated by Model 1 are improved by 3.2%, 4.42%, 5.72% separately. Moreover, it shows that Model 1 has higher TDE estimation accuracy than the conventional one, which illustrates that Model 1 has better traceability for the TDE of the MEMS accelerometer. In addition, the outputs of the MEMS accelerometer compensated by Model 2 are optimized up to 15.06%, 14.36% and 12.99% and higher than those compensated by the conventional model after introducing GA, which means that the performance of the novel model based on GA-BPNN is much better than the conventional model. The outputs of the MEMS accelerometer compensated by Model 3 are increased further by 16.01%, 15.47% and 15.97%, respectively, and higher than those compensated by the conventional model after introducing PSO-GA, which means the performance of the novel model based on PSO-GA-BPNN is much better than that of the novel model based on GA-BPNN. To demonstrate the performance improvement in TDE estimation real time of PSO-GA-BPNN, Table 2 shows MSEs between them at different iterations.

As shown in Figure 9d, when Model 2 and Model 3 go through the same iterations, the MSEs of Model 3 are smaller than those of Model 2. For less iterations, the convergence rate is much smaller than that of Model 2 in terms of quickly stabilizing the MEMS accelerometer. As they iterate more, the MSEs of Model 3 are close to those of Model 2. According to Table 2, the MSEs of Model 2 and Model 3 gradually approach the same after 50 iterations, and they have the same convergence at the same output accuracy and their real time is nearly the same. However, the MSEs of Model 3 are much smaller than those of Model 2 at 28 iterations and PSO reduces the iterations of GA-BPNN by 99.86%, which demonstrates that Model 3 has faster convergence to obtain better real time. Hence, the novel model based on the all-new TCQ and PSO-GA-BPNN has much better implementation to obtain higher TDE estimation accuracy and fewer iterations to increase its estimation real time.

## 4. Conclusions

In this paper, a novel TDE precise estimation model for a MEMS accelerometer using microstructure thermal analysis was proposed. By analyzing the microstructure thermal deformation at different ambient temperatures, qualitatively and quantitatively, TCQ (*T*, *T*^2^, ∆*T*, ∆*T*^2^) for TDE is studied clearly to obtain more accurate TDE traceability, which establishes a novel TDE precise estimation model for the MEMS accelerometer. Then, PSO-GA-BPNN was studied and applied in its parameter identification, which eliminated the local optimums in the conventional model based on BPNN to enhance TDE estimation accuracy and reduce BPNN’s probabilistic disorder to increase its real time. An all-new TDE test method was formed by analyzing heat conduction processes between MEMS accelerometers and thermal chambers, and a temperature experiment was designed with a proper temperature control interval and temperature control period. MSE was used to effectively evaluate TDE estimation accuracy and real time. The accuracy of the novel model based on TCQ and PSO-GA-BPNN was improved by 16.01% compared with the conventional one, and its iterations were reduced by 99.86% compared to the model based on GA-BPNN at maximum. The novel TDE precise estimation model for a MEMS accelerometer has the merits of higher TDE estimation and better real time, and decouples temperature dependence of Si-based materials, significantly improving the environmental adaptability of the MEMS accelerometer to expand its application in diverse complex conditions. Moreover, it is reliable and universal, so can be applied widely.

## Figures and Tables

**Table 1:** Performance comparison between the conventional and novel models.

	Test 1	Test 2	Test 3
Conventional model	8.43 × 10^−6^	1.81 × 10^−6^	9.62 × 10^−6^
Model 1	8.16 × 10^−6^	1.73 × 10^−6^	9.07 × 10^−6^
Model 2	7.16 × 10^−6^	1.55 × 10^−6^	8.37 × 10^−6^
Model 3	7.08 × 10^−6^	1.53 × 10^−6^	8.17 × 10^−6^
*Q*_1_	3.20%	4.42%	5.72%
*Q*_2_	15.06%	14.36%	12.99%
*Q*_3_	16.01%	15.47%	15.07%

**Table 2:** MSEs at different iterations.

Iteration	Model 2	Model 3	Iteration Effect
14	1.51 × 10^−^^3^	1.17 × 10^−^^4^	92.25%
28	6.50 × 10^−^^4^	7.40 × 10^−6^	99.86%
42	2.62 × 10^−^^5^	7.26 × 10^−6^	72.30%
56	7.24 × 10^−6^	7.17 × 10^−6^	0.90%
70	7.16 × 10^−6^	7.08 × 10^−6^	1.11%
